# Potential pathways for CO_2_ utilization in sustainable aviation fuel synthesis

**DOI:** 10.1039/d4sc06164k

**Published:** 2024-11-25

**Authors:** Enrique V. Ramos-Fernandez, Jose L. Santos, Dina K. Alsaadi, Anastasiya Bavykina, Jean Marcel R. Gallo, Jorge Gascon

**Affiliations:** a KAUST Catalysis Center (KCC), King Abdullah University of Science and Technology (KAUST) Thuwal 23955 Saudi Arabia jorge.gascon@kaust.edu.sa; b Laboratorio de Materiales Avanzados, Departamento de Química Inorgánica, Instituto Universitario de Materiales de Alicante, Universidad de Alicante Apartado 99 Alicante E-03080 Spain

## Abstract

The development of sustainable aviation fuels (SAFs) is a must for the decarbonization of the aviation industry. This paper explores various pathways for SAF production, focusing on innovative catalytic processes for the utilization of CO_2_ as a potential feedstock. Key pathways analyzed include the Modified Fischer–Tropsch Synthesis (MFTS), methanol synthesis, and subsequent transformations of methanol into hydrocarbons (MTH), aromatics (MTA) and olefin oligomerization. The potential of these processes is highlighted, alongside the challenges in catalyst development. The paper emphasizes the need for advanced catalytic processes to achieve high selectivity and stability under industrial conditions, which are critical for the commercial viability of CO_2_-based SAF production. Ultimately, this work aims to provide a comprehensive overview of the current state of research in SAFs, outlining promising directions for future research.

## Abstract 2

The development of sustainable aviation fuels (SAFs) is a must for the decarbonization of the aviation industry.

## Graphical abstract

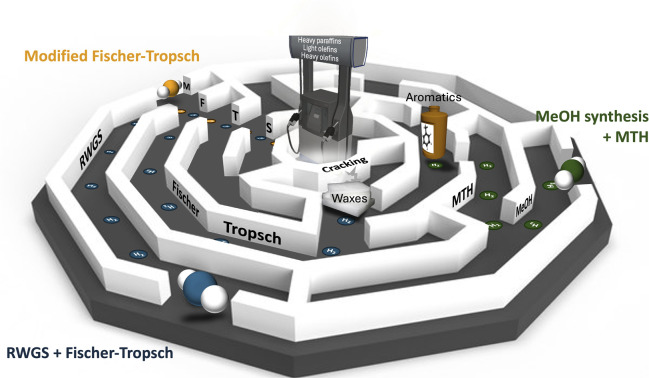

## Introduction

The aviation industry stands as a cornerstone in the fabric of the global economy, facilitating international trade, driving economic growth, and bridging cultures across vast distances. Its role is irreplaceable in fostering international relations and business development on a worldwide scale. However, this connectivity and dynamism come at a considerable environmental cost. As the world faces unprecedented challenges related to climate change, the aviation sector's significant contribution to greenhouse gas (GHG) emissions has become a critical focus. Recent reports indicate that the industry is responsible for more than 2% of all anthropogenic carbon dioxide (CO_2_) emissions globally, a figure that underscores the urgent need for a transition to more sustainable solutions.^1^

The quest for environmentally less harmful alternatives has brought the aviation industry to a turning point, where innovation and sustainability must converge to ensure its long-term viability. Against this backdrop, Sustainable Aviation Fuels (SAFs) emerge as a promising solution capable of drastically reducing the sector's GHG emissions and contributing to climate change mitigation. However, the development and implementation of these fuels face significant challenges, from technological and economic limitations to complexities in the supply chain.^2,3^

SAFs encompasses a range of non-fossil-based fuels that can significantly reduce the carbon footprint of air travel when compared to conventional jet fuels. Derived from sustainable resources such as waste oils, agricultural residues, and even CO_2_ captured from the air, SAF presents a promising avenue for reducing the aviation industry's greenhouse gas (GHG) emissions.

One of the key advantages of SAFs is their compatibility with existing aircraft engines and fuel infrastructure, enabling a seamless transition from traditional fuels without the need of extensive modifications to aircraft or refueling systems. This compatibility underscores SAF's role as a practical and immediately implementable solution to a long-standing problem.

The benefits of SAF extend beyond its renewable origins. The lifecycle GHG emissions of SAF, from production to combustion, are significantly lower than those of conventional jet fuels. Depending on the feedstock and production method, SAF can offer a reduction in carbon emissions of up to 80% compared to fossil jet fuel over its lifecycle.^1,2,4–7^ Moreover, SAF can also contribute to reducing the emission of pollutants such as sulfur oxides (SO_*x*_) and particulate matter, further enhancing air quality and contributing to a healthier environment.

The adoption of SAF is further motivated by regulatory pressures and the aviation industry's commitment to achieving carbon-neutral growth. Initiatives such as the Carbon Offsetting and Reduction Scheme for International Aviation (CORSIA) highlight the sector's dedication to environmental stewardship and underscore the critical role of SAF in meeting these ambitious goals.^7^

Despite the clear environmental and regulatory incentives, the widespread adoption of SAFs faces challenges, including limited feedstock availability, high production costs, and the need for further technological advancements. Overcoming these hurdles requires a concerted effort from governments, the aviation industry, fuel producers, and other stakeholders to invest in research and development, scale up production capacities, and create economic policies that incentivize the use of SAF.

## Not all long hydrocarbons are SAFs

The aim of this paper is not to detail all the regulations or characteristics of these fuels. Instead, we will highlight the most important ones to help the reader grasp the complexity of the challenge in preparing SAFs, particularly those derived from CO_2_.

Jet fuel specifications are critical to ensure performance, operability, and compatibility with existing aviation infrastructure. These standards are primarily defined by three ASTM norms: ASTM D1655, which specifies the requirements for conventional aviation turbine fuels; ASTM D4054, which provides a framework for the evaluation and approval of new aviation turbine fuels and fuel additives; and ASTM D7566, which applies to aviation turbine fuels containing synthetic hydrocarbons and is essential for sustainable aviation fuels (SAFs). Jet fuels must adhere to strict performance, operability, and drop-in requirements. Performance properties add value to the fuel in the context of a mission and include specific energy MJ kg^−1^, which is crucial for fuel efficiency by reducing takeoff weight; energy density 43.5–42.9 MJ L^−1^, important for volume-limited missions or military operations; emissions, including particulate matter, which affects environmental impact; and thermal stability, which ensures the fuel can resist degradation or coking under thermal stress, maintaining engine efficiency and longevity.

Operability properties are critical for the safe use of fuel under various engine conditions and include viscosity, important for flow performance, especially at cold temperatures; density, used in calculating fuel tank volumes; freeze point, preventing the fuel from freezing at high altitudes; flash point, ensuring safe handling and storage of the fuel; distillate temperature, ensuring sufficient volatility for efficient combustion; derived cetane number (DCN), important for stability during lean blowout limits; and minimum aromatic concentration, which ensures proper swelling of certain seals and O-rings previously exposed to high aromatic content fuels.

Drop-in requirements ensure the fuel can be used seamlessly with existing aircraft and infrastructure without modifications, meaning drop-in fuels must meet all performance and operability specifications and be fully fungible.

Jet fuel is composed of several hydrocarbon classes, each contributing to the fuel's overall performance. The typical composition includes *n*-alkanes (*n*-paraffins) at 20–26% by weight, iso-alkanes (iso-paraffins) at 30–37% by weight, monocyclic alkanes (cycloalkanes) at 19–25% by weight, bicyclic alkanes at 3–7% by weight, and aromatics at 14–19% by weight. These hydrocarbons are distributed across carbon numbers ranging from C_8_ to C_16_, with an average molecular weight typically around 152–166 g mol^−1^ depending on the specific jet fuel type.^8–11^

*n*-Alkanes, or *n*-paraffins, contribute significantly to the specific energy of the fuel, which is critical for efficient energy conversion in jet engines. However, larger *n*-alkanes tend to have high freeze points, making them less suitable for cold weather performance. Conversely, smaller *n*-alkanes might not meet the necessary flash point specifications.

Iso-alkanes, or iso-paraffins, are characterized by their branching structures, which result in lower freeze points compared to *n*-alkanes, enhancing the fuel's performance in cold conditions. Additionally, iso-alkanes offer better thermal stability, crucial for maintaining engine performance and preventing fuel breakdown at high temperatures. While iso-alkanes generally have lower energy density compared to aromatics, their specific energy is high, making them an essential component for efficient jet fuel.

Overall, paraffins (both *n*- and iso-) are critical in achieving a balance between energy content, cold-weather performance, and thermal stability. The specific properties of these hydrocarbons make them indispensable in formulating jet fuels that meet rigorous aviation standards. For instance, while *n*-alkanes contribute to the specific energy, iso-alkanes enhance low-temperature operability and thermal stability.

Cycloalkanes, or naphthenes, are integral components of jet fuel due to their cyclic molecular structures, which provide high energy density and superior thermal stability. These properties are essential for maintaining optimal performance in jet engines under high-temperature conditions without causing fuel degradation. Furthermore, cycloalkanes have relatively low freezing points compared to *n*-alkanes, which enhances the fuel's performance in cold environments, ensuring reliable operation at high altitudes.

On the other hand, aromatics are crucial for ensuring seal compatibility in fuel systems, particularly in engines that have historically used high-aromatic-content fuels. Despite their lower specific energy and higher sooting tendency during combustion, aromatics contribute to achieving the necessary fuel density and aid in maintaining the structural integrity of seals within the fuel system. However, the higher sooting tendency of aromatics necessitates careful balancing with other hydrocarbons to minimize particulate emissions and ensure cleaner combustion.

Theoretically, all these compounds can be prepared by hydrogenation with CO_2_ in one or several steps. The conversion of CO_2_ into SAFs is evolving and new lines of research are emerging. This work discusses potential avenues for future investigation, including the development of alternative catalyst systems, and process optimization strategies. Collaborative efforts across the scientific community, industry stakeholders, and policymakers will be essential to address the remaining challenges and to realize the potential of CO_2_-derived SAF in contributing to the sustainability of aviation and to broader climate change mitigation efforts.

According to ASTM D1655 and D7566 oxygenates in aviation fuel must be present at minimal levels. Generally, SAFs may contain various oxygenated species, making it necessary to subject them to a deoxygenation process. These oxygenated compounds can be a byproduct of SAF synthesis. Most of them can be generated during modified FT synthesis, although their production is both kinetically and thermodynamically hindered.^12,13^

In sum, the conversion of CO_2_ to SAF offers a promising solution to one of the aviation industry's most pressing challenges. This work aims to provide a comprehensive overview of the current state of research, the challenges ahead, and the potential pathways to a more sustainable aviation future, highlighting the role of cutting-edge catalytic chemistry in transforming environmental liabilities into assets.

## From CO_2_ to hydrocarbons

**Scheme 1:**
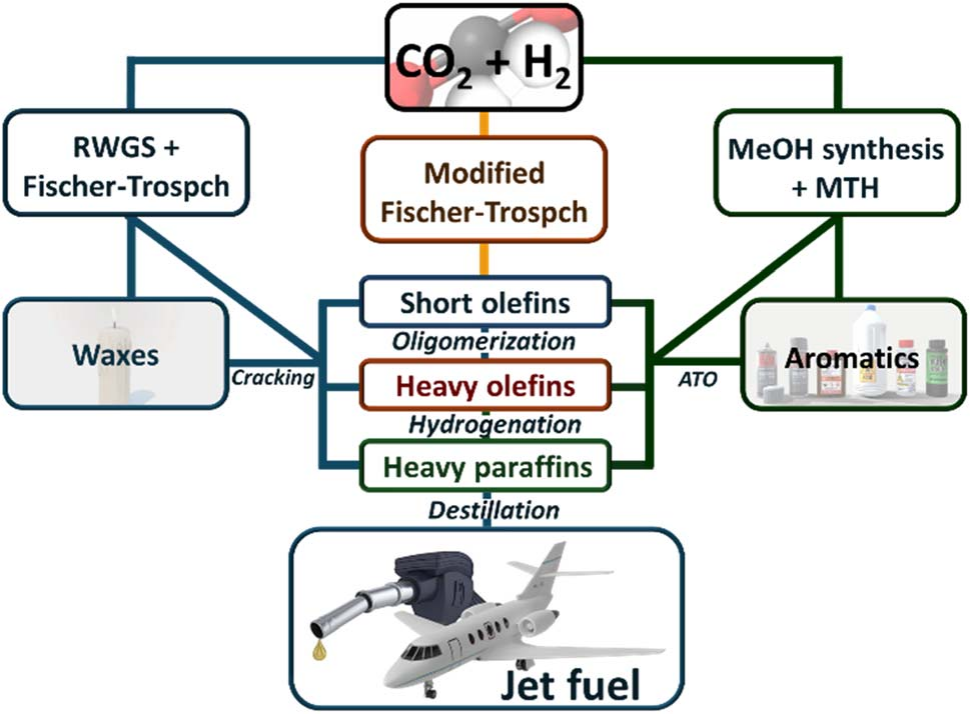
Possible routes for CO_2_ to be transformed into SAFs.

The hydrogenation of CO_2_ to produce valuable hydrocarbons is a promising pathway for converting greenhouse gas emissions into useful chemical feedstocks, offering a strategic approach to mitigate climate change while providing economic benefits. This process can follow three primary pathways (see Scheme 1).

The first pathway involves a two-step process where CO_2_ is first converted into carbon monoxide (CO) *via* the Reverse Water Gas Shift (RWGS) reaction.^14–20^ In the second step, the CO is then converted into hydrocarbons through Fischer–Tropsch Synthesis (FTS),^21–29^ which facilitates chain propagation to synthesize longer hydrocarbons. Both the RWGS reaction and the Fischer–Tropsch process have been extensively reviewed and are considered mature technologies, which fall outside the scope of this review.^30^

The second pathway integrates the RWGS and FTS into a single step, directly converting CO_2_ into hydrocarbons within the Sustainable Aviation Fuel (SAF) range. This approach simplifies the process by combining both reactions, making it more efficient and potentially more economical.^31^

Alternatively, the methanol-mediated synthesis pathway begins with the hydrogenation of CO_2_ to methanol. This initial step is followed by the conversion of methanol into hydrocarbons through processes such as the methanol to olefins (MTO) or others. Similar to MFTS, this route can generate a variety of hydrocarbons, with the specific outcomes largely dependent on the catalysts and reaction conditions tailored to optimize the conversion of methanol.^32–34^

All of these processes share a common challenge: the complexity of designing an efficient catalyst. When we examine the Modified Fischer–Tropsch Synthesis (MFTS), we see that the literature is quite limited, with most catalysts suffering from low selectivity and durability. A similar situation is observed in the methanol-mediated synthesis pathway. Although the initial step of methanol synthesis from CO_2_ is a more mature technology (which will be reviewed in detail below), the subsequent synthesis of hydrocarbons, especially those necessary for Sustainable Aviation Fuels (SAFs), remains underdeveloped.

In this review, we will focus on the catalysts used for these reactions and the advances that have been made or are needed to enable the effective synthesis of SAFs or SAFs precursors from CO_2_. Our goal is to explore the current state of catalyst development and identify gaps in the research.

### Modified Fischer–Tropsch synthesis

**Fig. 1:**
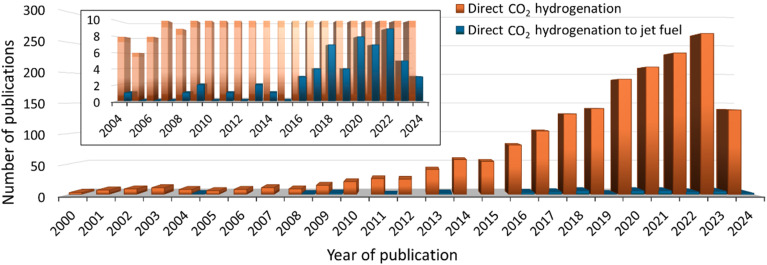
The number of publications related to direct CO_2_ hydrogenation to produce value-added chemicals (orange columns) and for the specific production of jet fuel (blue columns) in the last 24 years.

In the last 24 years, there has been exponential growth in papers related to the direct hydrogenation of CO_2_ to produce light olefins,^35–37^ liquid fuel hydrocarbons (such as gasoline, C_5_–C_12_,^38^ jet fuel, C_8_–C_16_,^39^ and diesel, C_12_–C_21_),^40,41^ and aromatics.^42^ However, from the 1800 publication, only 2% are dedicated to obtaining liquid hydrocarbons in the jet fuel range (C_8_–C_16_) (see Fig. 1). One of the major challenges for jet fuel production is designing catalysts that enable the production of hydrocarbons with the desired carbon chain length.^43^

#### The mechanism for CO_2_-based Fischer–Tropsch reaction

**Fig. 2:**
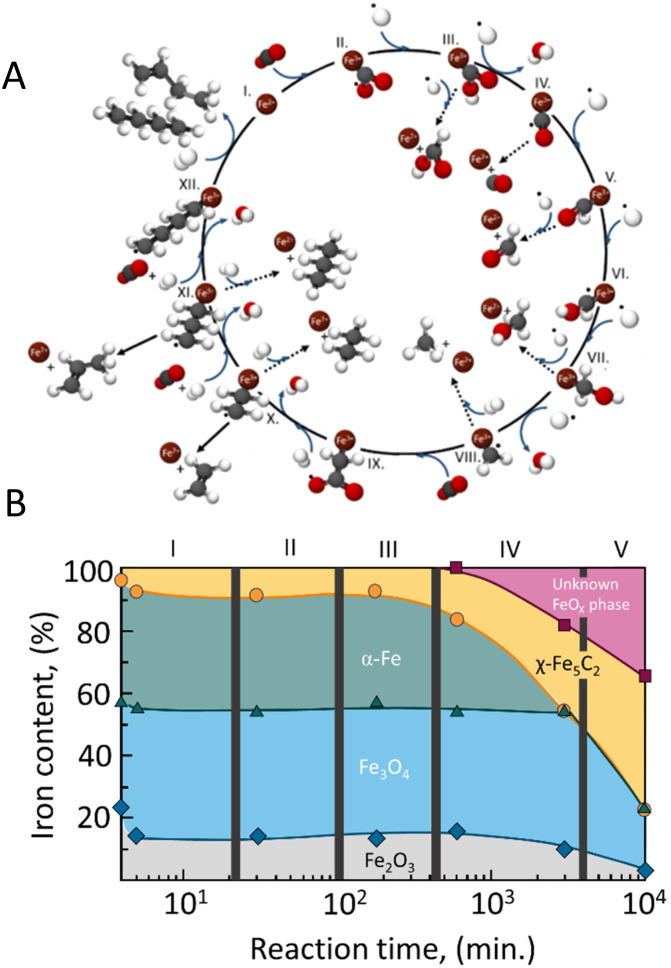
(A) Proposed overall reaction pathway of CO_2_ hydrogenation over iron-based catalysts. Adapted from ref. 44; and (B) iron-phase composition as a function of time during Fischer–Tropsch synthesis determined by Mössbauer spectroscopy over a Fe–Cu/K/Al catalyst. Adapted from ref. 45.

It is widely accepted that the reaction pathway for the single step begins with the reduction of CO_2_ to CO through the reverse water–gas shift reaction. For instance, in the case of Fe catalysts, CO_2_ is activated by iron(ii) which reacts with H* to form carboxyl (Fig. 2A, steps I–III). Further reduction takes place, leading either to the desorption of formic acid or the formation of CO (step IV). The reduction of carbon monoxide to methylene (*CH_2_) takes place by consecutive hydrogenation steps (steps V–VIII) forming formate (*CH_2_O) and hydroxymethyl (*CH_2_OH) intermediates and, hence, desorption of formaldehyde, methanol, and methane are expected, although undesirable.^44,45^

The chain propagation steps are more debated. For example, in the case of iron-based catalysts, it has been proposed that the reaction between CO_2_ and the methylene species produces 
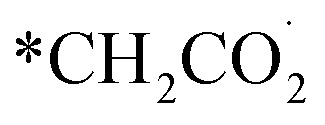
, which is then reduced to an alkyl group, enabling consecutive propagation (Fig. 2A, steps IX–XII).^45^ Alternatively, the carbide mechanism has also been proposed, and, in this case, carbon monoxide is dissociated into C* and O*, and these species are hydrogenated to *CH_2_ and H_2_O(g). The methylene species formed on the surface undergo a coupling reaction, leading to carbon-chain growth.^46,47^ In direct CO_2_ hydrogenation to jet fuel over an iron-based catalyst, the chain propagation for α-olefin formation is considered a preferential pathway due to the low H/C ratios used, which minimize the complete hydrogenation of radicals, thus favoring the production of olefins over paraffins.^48,49^

Considerable research has focused on Fe-based catalysts for CO_2_-FTS. The catalyst development required optimizing the correlation between C–O activation and C–C coupling reactions. As shown in Fig. 2A, Fe-based catalysts can produce CO, methane, and methanol. Hence, obtaining the Fe active phases that promote adequate hydrocarbon chain growth and minimize the C_1_–C_4_ products are major challenges for jet fuel production.

Riedel *et al.*^45^ have demonstrated that understanding the structural changes of Fischer–Tropsch iron catalysts under reaction conditions, as well as the pretreatment procedures, is essential for designing optimal catalysts. The authors propose that the transition to a steady state in Fischer–Tropsch synthesis with iron can be divided into distinct stages, as illustrated in Fig. 2B.

Initially, the fresh catalyst, composed of magnetite (Fe_2_O_3_) and alkali, shows minimal activity for both the water–gas shift reaction and FTS. However, as the reaction progresses, the Fe_3_O_4_ and Fe_2_O_3_ phases are converted into an oxidic iron phase that is active for the (reverse) water–gas shift reaction. Over time, Fischer–Tropsch activity emerges as the α-Fe reacts with carbon from CO dissociation, converting the iron into iron carbide, particularly Fe_5_C_2_, which is suggested to be the actual active site for FTS.

Zhang *et al.*^50^ investigated the structural evolution and performance of iron catalysts throughout their entire life cycle in CO_2_ hydrogenation to produce C_2_–C_4_ olefins. Using *operando* techniques and *ex situ* characterization, the study showed how the catalyst undergoes phase transitions during the stages of activation, reaction, deactivation, and regeneration. During activation, the iron catalyst transitions from Fe_2_O_3_ to χ-Fe_5_C_2_ (Hägg carbide), identified as the key active phase. During the reaction, this Fe_5_C_2_ phase is initially stable, but as the reaction progresses, it irreversibly converts to Fe_3_O_4_, leading to a significant decrease in catalytic activity and olefin selectivity. Additionally, Fe_3_C phases and carbon deposits are found in the deactivated catalyst. Regeneration methods are explored, with a combined CO_2_–CO treatment proving to be the most effective, recovering up to 91.8% of the original activity and 97.7% of the original selectivity. However, the oxidation of Fe_5_C_2_ is identified as the primary deactivation mechanism, while carbon deposition and sintering have a comparatively minor impact.

Although iron was initially proposed as the active phase in direct CO_2_ hydrogenation due to its superior ability for chain growth, stability, and lower activity for the WGS reaction, cobalt-based catalysts have also been extensively utilized for jet fuel production. Cobalt metal is active in the FTS process but is less effective in promoting the RWGS reaction because of its high activity toward methanation. There have been limited studies on the precise mechanism of CO_2_ hydrogenation to jet fuel using cobalt-based catalysts, primarily due to low C_5+_ selectivity and different preferred reaction pathways. A possible reaction scheme has been proposed for the core–shell Co@CoO_*x*_/Co_2_C catalyst.^51^ In this scheme, the oxygen-vacant CoO_*x*_ in the outer shell promotes the RWGS reaction, and the resulting CO is transported to the Co_2_C phase and metallic Co center in the core, where it produces long-chain C_5+_ hydrocarbons *via* the FT reaction. For Fe–Co-based catalysts, it has been suggested that chain propagation occurs through the interaction between the alkoxide-activated Co site and iron-activated *CO. Additionally, cobalt can be associated with Cu or Mo, and alkali metals may be introduced to enhance CO_2_ reduction to CO and inhibit the undesired CO_2_ hydrogenation to CH_4_.^52^

#### Product distribution

Hydrocarbon products from CO-FT synthesis follow the Anderson–Schulz–Flory (ASF) distribution, which is inherently broad and lacks selectivity. The relationship between hydrocarbon product selectivity and the probability of chain growth is expressed by eqn (1) and (2):

1 *α* = *r*_p_/(*r*_p_ + *r*_t_)

2 *W*_*n*_ = *n*(1 − *α*)^2^ × *α*^*n*−1^

**Fig. 3:**
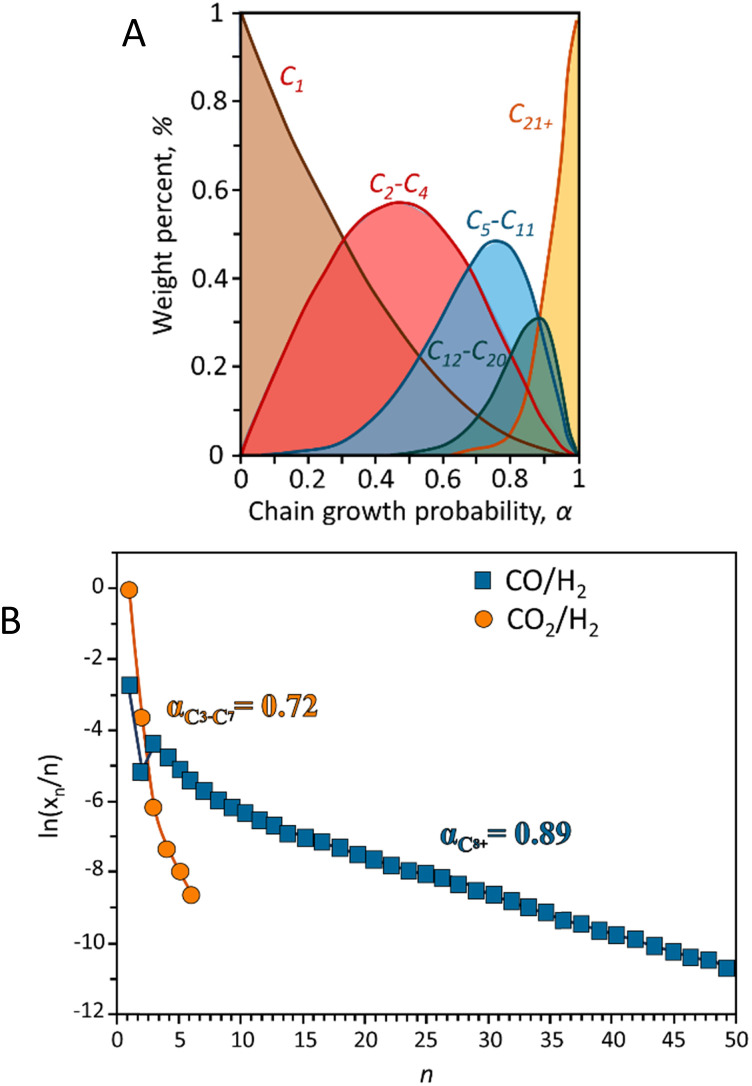
(A) Hydrocarbon distribution predicted by ASF model. Adapted from ref. 53 and (B) hydrocarbons experimental ASF plots reported in terms of selectivity during CO and CO_2_ hydrogenation. Adapted from ref. 54.

Here, *n* represents the carbon number (*n* > 1), and *α* is the chain growth probability. The value of *α* is considered independent of *n* and is determined by the rates of chain propagation (*r*_p_) and termination (*r*_t_). Eqn (2) provides additional mathematical expressions, where *W*_*n*_ is the weight fraction of C_*n*_ products. Fig. 3A illustrates the selectivity of hydrocarbons for different *α* values, with maximum selectivities for C_2_–C_4_, C_5_–C_11_, and C_8_–C_16_ hydrocarbons being 58%, 48%, and 41% by weight, respectively.^53,54^

Much research has been focused on developing various strategies to improve the selectivity of target hydrocarbons beyond the maximum predicted by the ASF model. For instance, introducing small amounts of α-olefins (like 1-octene, 1-decene, or 1-tetradecene) as a co-feed with syngas can increase the selectivity for jet fuel (C_8_–C_16_ hydrocarbons) to over 65% in FTS reactions. However, this method is less effective for the direct hydrogenation of CO_2_ to α-olefins. An enhanced product-distribution model for bifunctional catalytic systems including a cracking degree parameter (*β*) to better describe the selectivity for jet fuels has been described,^55^ although this model does not accurately predict selectivity across the full spectrum of hydrocarbons, eqn (3).^53,56^3
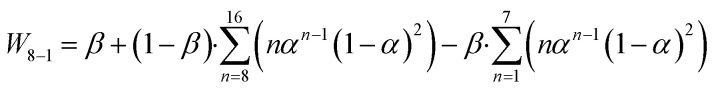


In CO_2_ hydrogenation, the selectivity for α-olefins is constrained by limited chain growth reactions on iron carbide surfaces. To overcome this, introducing additional active sites for olefin re-adsorption could enhance oligomerization and improve jet fuel formation. To highlight the differences in product distributions between CO and CO_2_ hydrogenation, Fig. 3B presents typical Anderson–Schulz–Flory (ASF) diagrams illustrating the selectivity of total hydrocarbons in both processes. Unlike CO hydrogenation, CO_2_ hydrogenation does not follow a typical ASF distribution. The low carbon-to-hydrogen ratio observed in CO_2_ hydrogenation results from the sluggish adsorption rate of CO_2_ on the catalyst surface. This process favors the hydrogenation of intermediates adsorbed on the surface, which enhances methane production while limiting the growth of longer carbon chains. For specific catalytic systems the inherent kinetic limitations are often linked to thermodynamic constraints.^54,57^ The thermodynamics RWGS imposes significant limitations on both the reaction rate and selectivity in CO_2_ hydrogenation. Achieving chain-propagation probabilities similar to those in CO hydrogenation requires decoupling chain-growth kinetics from gas-phase CO concentration. This can be accomplished using iron carbide-based Fischer–Tropsch catalysts, where molecular CO coverage remains low during operation. As a result, CO presence is less critical for promoting chain growth, reducing the premature desorption of products as light hydrocarbons. Further investigation is needed to develop a statistical distribution model for specific products.

#### Fe- and Co-based catalysts for the single-step conversion of CO_2_ to jet fuel

The direct hydrogenation of CO_2_ to jet fuel has primarily been explored using traditional Fischer–Tropsch synthesis (FTS) catalysts, such as iron (Fe) and cobalt (Co), due to their high activity, selectivity, and stability.^58^ There are also a few studies involving other metals. For example, Ru-based catalysts have demonstrated activity in converting CO_2_ to jet fuel at 180 °C, but they tend to achieve high selectivity for methane.^59,60^ This discussion will focus on Fe and Co-based catalysts, where the formation of significant concentrations of jet fuel range hydrocarbons (C_8_–C_16_) has been confirmed. Table 1 provides details on catalyst composition, reaction conditions, and catalytic performance.

**Table 1:** Reaction conditions and catalytic performances of Fe and Co-based catalysis for the single-step conversion of CO_2_ to jet fuel

Catalyst	Reaction conditions				CO_2_ conversion (%)	Selectivity (%)				C_5+_ yield (%)	Ref.
	Temperature (°C)	Pressure (MPa)	GHSV (mL g^−1^ h^−1^)	H_2_/CO_2_ molar ratio		CO	CH_4_	C_2_–C_4_	C_5+_		
Fe–K/AI_2_O_3_ (15.7Fe : 5.5K : 78.7Al_2_O_3_ wt%)	300	1	1000	3	40.8	11.1	7.4	25.8	55.7	22.7	44
Fe–Cu–Al–K (77.9Fe : 6.5Cu : 9.4Al : 6.2K)	265	1	2000	3	37.1	9.1	7.0	23.2	60.7	22.5	61
Fe–Cu from delafossite (CuFeO_2_)	300	1	1800	3	16.7	31.4	1.6	22.4	44.5	7.4	40
Fe–Cu–Al–K	265	1.3	2240	3	15.6	22.8	9.9	27.8	39.4	6.1	62
Zn–Fe–K	320	2		2.5	47.1	8.7	14.8	46.1	46.1	21.7	63
Zn–Fe–Na	320	2		2.5	46.7	9.2	15.0	51.0	34.0	15.9	63
Zn–Fe–K	320	2		2.5	47.1	8.7	14.8	46.1	46.1	21.7	63
Fe–Zn–Co–K	320	2		2.5	50.2	8.1	—	—	57.8	26.7	47
ZnFe_2_O_4_ [0.08 wt% Na]	340	1	1800	3	34.0	11.7	9.7	31.8	58.5	19.9	41
Fe–Mn–K	300	1	2400	3	38.1	5.6	9.8	26.1	58.4	22.3	39
Co@CoO_*x*_/Co_2_C	270	4	4000	3	64.3	0.2	44.1	22.9	32.8	21.1	51
K–CoCu/TiO_2_ (Co/Co molar ratio 1.4)	250	5	3000	3	13	35.1	22.1	20	22.8	3.0	64
Na–CoCu/TiO_2_ (Co/Co molar ratio 1.7)	250	5	3000	3	18.4	30.2	18.2	22.2	29.4	5.4	64
CoFeNa (2Co : 1Fe : 0.81Na)	240	3	5500	3	10.2	5.2	17.8	9.4	72.9	7.4	65

**Fig. 4:**
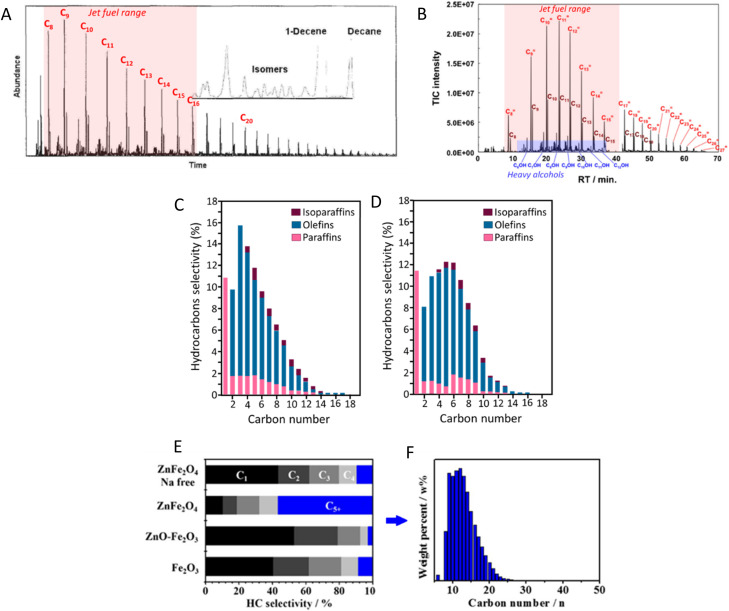
GC-MS total ion chromatogram of liquid products collected from CO_2_ hydrogenation on (A) Fe–K/Al_2_O_3_ and (B) FeCo–K/Al_2_O_3_ catalysts. Adapted from ref. 44 and 65, respectively. Detailed hydrocarbon distribution over (C) Zn–Fe–K and (D) Zn–Fe–Co–K catalysts. Adapted from ref. 47. CO-free hydrocarbon selectivity of (E) Fe_2_O_3_, ZnO–Fe_2_O_3_, ZnFe_2_O_4_, and Na-free ZnFe_2_O_4_ catalysts, and (F) carbon-number distribution of the liquid products of CO_2_ hydrogenation with the ZnFe_2_O_4_-derived catalyst, as determined by a simulated distillation method (ASTM D2887). Adapted from ref. 41.

In the early 2000s, Fe–K/Al_2_O_3_ was demonstrated to effectively promote the direct conversion of CO_2_ to C_5+_ hydrocarbons with a yield of approximately 22%, which is among the highest yields reported for jet fuel production (Table 1, entry 1). As shown in Fig. 4A, detailed GC analysis of the product obtained using Fe–K/Al_2_O_3_ reveals a predominance of hydrocarbons in the jet fuel range (C_8_–C_16_), with a peak at C_9_ compounds. A significant challenge during the early stages of catalyst development was minimizing methane production, which was addressed by impregnating the catalyst with potassium.^61^ Indeed, as noted in Table 1, most catalysts developed for CO_2_-based Fischer–Tropsch synthesis incorporate either K or Na. Generally, the addition of alkali promoters significantly enhances jet fuel production by improving the catalyst's properties and performance. Although the role of potassium is not fully understood, promoters typically act as electronic or structural enhancers, or both, boosting catalyst performance in C–O activation and C–C coupling reactions. Structural promoters influence the formation and stabilization of the catalyst's active phase, improving dispersion and often leading to higher activity. On the other hand, electronic promoters affect the electron density near the catalyst's valence band, altering the local electron density on the surface and consequently modifying the active sites.^66^

It has been suggested that potassium acts as an electronic promoter in direct CO_2_ hydrogenation rather than as a structural promoter, thereby regulating the phase proportions of Fe_0_/Fe_*x*_O_*y*_/Fe_*x*_C_*y*_ to maintain an optimal balance. Potassium lowers the metal work function by donating electron density to the vacant d orbitals of iron, which enhances CO dissociation while reducing H_2_ adsorption. This reduction in H_2_ adsorption decreases alkene hydrogenation, leading to a higher alkene content. Although excessive potassium can poison iron catalysts in Fischer–Tropsch synthesis, in CO_2_ hydrogenation, a higher potassium content is actually beneficial.^51^

It increases CO_2_ conversion, reduces methane yield, and significantly enhances the alkene/alkane ratio, promoting the formation of longer-chain hydrocarbons.^67^

For example, carbonated potassium promoters such as K_2_CO_3_, CH_3_COOK, and KHCO_3_ enhance the formation of the χ-Fe_5_C_2_–K_2_CO_3_ interface during CO_2_ hydrogenation over Fe-based catalysts by facilitating the migration of the K promoter. CO_2_ initially reacts with K_2_CO_3_ to form KCOOH and CO, with the generated CO then participating in subsequent Fischer–Tropsch synthesis reactions to produce olefins. In contrast, non-carbonated potassium promoters are less effective in forming iron carbide species at the interface. Studies have shown that maintaining an optimal distance between K_2_CO_3_ and Fe species can increase CO_2_ conversion to 32% and olefin selectivity to nearly 75%, ensuring high catalytic stability. The Fe_2_O_3_@K_2_CO_3_ catalyst demonstrates exceptional performance, achieving a 44% CO_2_ conversion rate and 63% selectivity for C_2_–C_10_ olefins in CO_2_ hydrogenation.^68^

The spinel structure ZnFe_2_O_4_, impregnated with potassium, has achieved a 21.7% yield for C_5+_ hydrocarbons, which can be further enhanced to 26.7% by incorporating approximately 2 mol% of cobalt into the structure.^63^ It is suggested that ZnO functions as a structural promoter in CO_2_ hydrogenation for jet fuel synthesis by preventing the oxidation of Fe_5_C_2_ and modifying the Fe_*x*_C_*y*_/Fe_*x*_O_*y*_ ratio. This promotes CO_2_ and CO activation, improves C–C coupling, and regulates olefin desorption and hydrogenation, balancing the RWGS and FTS reactions.^69^ Moreover, cobalt regulation has been identified as crucial for efficiently converting CO_2_ to jet fuel by reinforcing RWGS and chain propagation reactions, shifting the product distribution through an oxygen-containing intermediate pathway (CO*, HCOO*, 
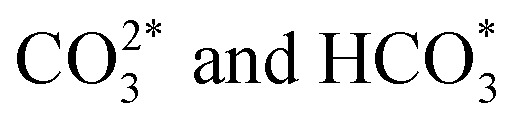
).^47^ Detailed hydrocarbon distribution analysis for ZnFe_2_O_4_, with and without cobalt, shows a significant presence of hydrocarbons below C_8_, which could be suitable for jet fuel applications, though further optimization is needed to maximize selectivity for C_8+_ products (Fig. 4C and D).

Interestingly, ZnFe_2_O_4_ synthesized using a microwave-assisted hydrothermal method and doped with 0.08 wt% of sodium achieved a high CO_2_ conversion to C_5+_ hydrocarbons with a 19.9% yield, producing mainly jet fuel range hydrocarbons (Fig. 4E and F).^41^ The differences in product distribution observed between spinel structure K–ZnFe_2_O_4_ and microwave-assisted synthesized Na–ZnFe_2_O_4_ highlight the significant influence of synthesis methodology on catalytic performance.^39^ An Fe–Mn–K catalyst was prepared using an organic combustion method, where the organic fuels used also acted as chelating agents, facilitating the formation of nanostructured materials. This catalyst achieved a 38.2% CO_2_ conversion and a 17.2% yield for C_5_–C_16_ hydrocarbons (22.3% yield for C_5+_). Additionally, the reaction produced light olefins (ethylene, propylene, butenes) with an 8.7% yield, which is valuable for the petrochemical industry and typically derived from fossil fuels.

Co-based catalysts, commonly used in industrial CO-FTS processes, have limited RWGS activity and generally require a second component for converting CO_2_ to CO. The limitations of cobalt-based catalysts for converting CO_2_ into liquid hydrocarbons stem from thermodynamic constraints that impact reaction kinetics. Under standard conditions, the RWGS equilibrium restricts the achievable CO partial pressure, which in turn limits both the overall CO_2_ conversion rate and chain propagation on cobalt catalysts.^57^ While cobalt-based catalysts typically achieve high chain-growth probabilities (*α* > 0.85) and C_5+_ selectivities (>80%) with H_2_/CO feed, the lower CO pressures under RWGS conditions reduce chain-growth probabilities to below 0.5–0.6, resulting in C_5+_ selectivities below 20% for H_2_/CO_2_ feeds.^54,57^ Indeed, these catalysts typically show high selectivity for CH_4_ and light hydrocarbons (>55%). However, the addition of transition metal promoters can enhance the selective formation of liquid hydrocarbons. For example, a Mn-promoted Co@CoO_*x*_/Co_2_C catalyst efficiently converted CO_2_ into liquid fuels and lube base oil in a single pass. In this system, oxygen vacancies in CoO_*x*_ activated RWGS, while the Co_2_C phase and metallic Co core drove FTS for long-chain hydrocarbons.^51^ This catalyst demonstrated remarkable stability, sustaining CO_2_ conversion for 1425 hours, with minimal impact from sintering and carbon deposition. However, the product distribution for C_5+_ hydrocarbons varied over time, with selectivity for C_5_–C_20_ hydrocarbons decreasing from 24.5% to 12.6%, while selectivity for C_21+_ increased from 6.5% to 14.9% over 1425 hours. At 270 °C, the catalyst achieved a 64.3% CO_2_ conversion with a 21.1% C_5+_ yield, and over 1425 hours, C_21+_ selectivity increased to 14.9%.

Na and K-doped CoCu/TiO_2_ catalysts promoted CO_2_ hydrogenation with a 3.0–5.2% yield to C_5+_ hydrocarbons, maintaining stability for over 200 hours on stream.^64^ The Na-modified catalyst showed the best performance due to sodium's strong basicity and minimal impact on H_2_ desorption. The positive effects of alkali promoters could guide the design and optimization of direct CO_2_ hydrogenation processes for jet fuel production. Indeed, a Co–Fe–Na catalyst derived from a layered double hydroxide was shown to promote C–C coupling and enable high C_8_–C_16_ selectivity (63.5%) while inhibiting the undesired CO_2_ methanation reaction. Despite a 72.9% selectivity for C_5+_, the low CO_2_ conversion resulted in a modest 7.4% C_5+_ yield.^65^

In Fe-based catalysts, the co-existence of alkali and alkaline-earth metals promotes the formation and stabilization of the iron carbide phase.^70^ For example, strontium (Sr) enhances the electronic interaction between sodium (Na) and iron (Fe) species, maintaining catalytic stability for over 500 hours of reaction. Sr acts as both a structural and electronic promoter, improving C–O dissociative adsorption and subsequent C–C coupling, thereby facilitating CO_2_ hydrogenation.

However, under CO_2_ hydrogenation conditions, Fe-based catalysts often experience deactivation, primarily due to the transformation of the active χ-Fe_5_C_2_ phase into Fe_3_C and Fe_3_O_4_,^50^ particularly near the reactor inlet.^71^ This deactivation occurs for two main reasons: (a) water produced as a byproduct, which, under hydrothermal conditions, oxidizes the χ-Fe_5_C_2_ phase, and (b) the modified CO_2_-FT process requires a significantly higher H_2_/C ratio than traditional CO-FT, making the FeC_*x*_ phases thermodynamically unstable. Another cause of catalyst deactivation is the formation of carbon deposits through the Boudouard reaction, which poisons the active sites.^71^ Although it has been shown that the Fe-active phase can be regenerated using mild oxidizers such as CO or CO_2_/CO, the carbon deposits tend to persist under these conditions.

Inspired by the exceptional performance of Fe–K for the one-step conversion of CO_2_ to jet fuel, other studies have explored the performance of Fe-based bimetallic catalysts. For example, Fe–Cu–Al–K achieved a selectivity for C_5+_ hydrocarbons comparable to that reported for Fe–K, but at twice the gas hourly space velocity (GHSV) (Table 1, entry 2).^61^ Later research revealed that copper enhances the catalyst's ability to adsorb CO_2_, dissociate C–O bonds, and promote chain growth by forming Hägg carbide (χ-Fe_5_C_2_) *in situ* during the reaction. A comparative study demonstrated that while Fe_2_O_3_ and a physical mixture of Fe_2_O_3_–CuO only achieved about 2% selectivity for C_5+_ hydrocarbons, significantly higher selectivities were observed for Fe–Cu catalysts derived from delafossite (44.5%) and coprecipitation (39.4%) (Table 1, entries 3 and 4).^40^ These findings highlight the importance of the close proximity between Fe and Cu sites in forming different active phases (χ-Fe_5_C_2_, Fe_3_O_4_), which greatly enhances jet fuel selectivity.^62^

**Fig. 5:**
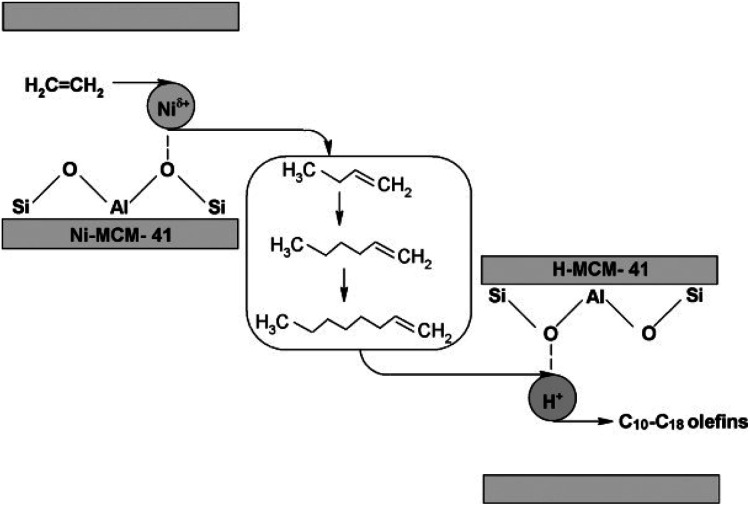
Schematic representation of consecutive oligomerization of ethylene. Reproduced with permission of ref. 73.

Bimetallic Fe–Co–K/Al_2_O_3_ has been reported to enhance hydrocarbon chain propagation while reducing methane selectivity.^72^ This process predominantly produces linear alpha olefins within the jet fuel range, with only trace amounts of alcohols. Notably, while Fe–K catalysts primarily yield a C_5+_ mixture with C_8_–C_10_ as the major components, Fe–Co–K catalysts extend the carbon chain, resulting in C_10_–C_12_ as the dominant hydrocarbons in the mixture (Fig. 5).

## Methanol mediated synthesis

### Methanol synthesis

Methanol is commercially manufactured from fossil fuels-derived syngas, predominantly obtained by natural gas reforming or coal gasification.^74^ The life-cycle CO_2_ emissions of the process account for approximately 10% of the emissions in the chemical and petrochemical industries.^75^ Envisioning the decarbonization of the chemical sector, e-methanol can be obtained from the hydrogenation of waste CO_2_ streams using green hydrogen. Hence, in the energy transition, methanol production shall utilize carbon dioxide instead of producing it. This paradigm shift associated with the versatility to produce hydrocarbons typically derived from oil (olefins, aromatics, and fuel-range hydrocarbons)^76–79^ shall place methanol at the centre of a future low-emissions chemical industry. Yet, producing methanol from CO_2_ requires further development, particularly regarding the catalyst activity to methanation and its stability in the presence of typical feedstock impurities (Table 2).^92^

**Table 2:** Methanol synthesis catalysts

Catalyst	H_2_ : CO_2_ ratio	Temp. (°C)	Pressure (MPa)	Space velocity (mL g^−1^ h^−1^)	*X*_CO_2__ (%)	*S*_CH_3_OH_ (%)	*Y*_CH_3_OH_ (%)	Space-time yield (g_MeOH_ g_cat_^−1^ h^−1^)	Ref.
Cu/Zn/Al (66/30/11)	3	220	2.8	1525	20.3	63.2	13.0	0.07	80
28.23 wt% CuZnO/SiO_2_	3	220	3	2000	14.1	57.2	8.0	0.06	81
CuO–ZnO–ZrO_2_ (1.0 wt% SiO_2_)	3	240	2	3900	5	70	3.5	0.24	82
Cu^2+^ : Zn^2+^ : Al^3+^ : Zr^4+^ (6 : 3 : 0.5 : 0.5)	3	270	5	4600	24.5	57.6	14.1	0.21	83
Cu/Zn/Al/Zr (52.5/24.9/17.1/5.5)	3	230	5	8500	19.3	58.5	11.3	0.33	84
Cu/Zn/La/Al (60/30/3/7)	4	300	8.5	55 000	10	65		0.79	85
In_2_O_3_/ZrO_2_	4	300	5	16 000		100		>0.4	86 and 87
InCo-2	4	300	5	22 000	16	83	13.3	0.83	88
13% ZnO–ZrO_2_	3	320	5	24 000	10	86			89
ZnZrO_*x*_	4	320	5	24 000	20 (approx.)	80 (approx.)		0.46	90
In@ZnZrO_*x*_	3	330	5		13.5	82.7		0.749	91

Below, we discuss the specifics of three major catalysts used in this process.

#### Cu/ZnO/Al_2_O_3_

Cu/ZnO/Al_2_O_3_ (CZA) catalyst is the industry standard for methanol synthesis due to its efficiency and robustness. Within this ternary system, copper provides the active sites for the reduction of CO_2_ to methanol, while zinc oxide acts as a stabilizer for these copper sites, preventing their rapid degradation. Alumina serves as a structural support that maintains the physical integrity of the catalyst particles under the high-temperature conditions typical of CO_2_ reactions.

Due to its high performance, various CZA formulations and preparation methods have been explored since its development by Imperial Chemical Industries.^93^ For instance, Ren *et al.* prepared CZA catalysts using the co-precipitation method.^80^ They investigated the effects of catalyst preparation conditions, reaction temperature, and pressure on methanol and DME synthesis. Catalyst performance was influenced by preparation conditions such as precursor concentration, reaction temperature, and pressure. Lower precursor concentrations resulted in higher surface areas and better dispersion. Optimal conditions for methanol synthesis were found at 220 °C and 2.8 MPa, yielding a methanol selectivity of 63.2%. Additional elements also proved to be beneficial for CZA. Phongamwong *et al.* investigated the impact of SiO_2_ content on CZA catalysts.^82^ Introducing SiO_2_ improved the catalysts' performance with 1 wt% SiO_2_ yielding the best results. This catalyst showed a 26% increase in methanol synthesis activity compared to the SiO_2_-free version. SiO_2_ enhanced metal dispersion and surface area, thus contributing to higher stability and methanol selectivity, highlighting the role of SiO_2_ as a structural promoter. Micro-spherical SiO_2_-supported Cu/ZnO in a slurry phase reactor was suggested by Jiang *et al.*^81^ Using the ammonia-evaporation method, catalysts with varying Cu and ZnO loadings were synthesized and tested. Optimal performance was achieved with a 28 wt% metal (Cu + ZnO) loading, showing high methanol selectivity and CO_2_ conversion due to small Cu particle size and uniform dispersion. However, further increases in metal loading blocked pores and reduced reducibility. The catalysts also demonstrated good stability over long-term use.

Dong *et al.*^83^ studied the liquid reduction method for catalyst preparation, As opposed to the more conventional co-precipitation method, liquid reduction synthesis method was proposed by Dong *et al.*,^83^ who also included Zr in the system. This approach led to the formation of Cu/Zn/Al/Zr catalysts containing Cu in three valence states (Cu^2+^, Cu^+^, Cu). The liquid reduction method allowed for better control over the particle size and distribution of Cu species, resulting in improved catalytic activity and stability. Another example is fluorinated Cu/Zn/Al/Zr hydrotalcite-derived nanocatalysts that were explored by Gao *et al.*^84^ By incorporating different amounts of fluorine into the hydrotalcite structure, the catalysts showed varied Cu particle sizes and surface areas. An optimal F/Al ratio of 0.83 resulted in high CH_3_OH selectivity and yield. The fluorine-modified catalysts demonstrated substantial stability and enhanced methanol production. Ali *et al.* modified copper-based catalysts with La_2_O_3_ by the solution combustion synthesis method.^85^ Incorporating 3 wt% La_2_O_3_ into CuO/ZnO/Al_2_O_3_ catalysts resulted in smaller metal oxide particles, increased surface defects, and more oxygen vacancies, leading to higher catalytic activity. At 300 °C, 85 bar, and a gas hourly space velocity of 55 000 h^−1^, the promoted catalyst achieved 20% conversion and 65% methanol selectivity, with a methanol production rate of 0.79 g g_cat_^−1^ h^−1^. The study attributes this high catalytic efficiency to the formation of Cu–LaO_*x*_ mixed oxide phases, improved dispersion of Cu nanoparticles, and the creation of moderately basic sites and oxygen vacancies.

#### InCo

Cu/ZnO/Al_2_O_3_ is the benchmark catalyst in the industry for methanol synthesis. Still, there exist some disadvantages of this system when a pure CO_2_ stream is used. Low stability, formation of undesired methane as a byproduct, and low methanol productivity are among them.

Indium cobalt-based catalysts are an area of active research as they represent an alternative to more traditional catalysts. The combination of indium and cobalt offers a different set of active sites and can potentially offer enhanced activity or selectivity for methanol production from CO_2_ and H_2_.

They might offer higher conversion rates, better tolerance to catalyst poisoning (a common issue with traditional catalysts), and improved selectivity in methanol production. Such traits could redefine industrial standards and open new avenues for more efficient and environmentally friendly catalyst designs.

Indium oxide supported on porous zirconia showed to facilitate oxygen vacancies formation.^86^ Bavykina *et al.* combined indium and cobalt oxides to boost this performance further. Cobalt oxide, a good methanation catalyst, was turned into a methanol production catalyst when combined with indium oxide.^88^ The synergetic effect of Co and In resulted in higher productivity of 0.86 g_CH_3_OH_ g_cat_^−1^ h^−1^ at temperatures up to 300 °C. The formation of higher amount of oxygen vacancies was confirmed and an amorphous layer containing CoO_*x*_ and In_2_O_3_ oxides was identified as an active phase. The original catalyst, denoted as InCo-2, was prepared by a facile coprecipitation method. Later, the same group reported another synthesis of InCo using indium doped Zn-based metal organic framework as a sacrificial agent.^93–95^

Compared to the CZA catalyst, InCo system offers higher conversions and methanol yields over a wide range of temperature. At temperatures above 300 °C InCo starts outperforming the classic benchmark CZA. This is an important feature that would benefit a more complex system where a methanol catalyst is combined with other system that are subsequently converting the produced methanol.^96^

#### ZnZrO_2_

The ZnCrO_*x*_ catalyst, developed by Mittasch *et al.*^97^ at BASF, was a significant advancement in methanol synthesis, enabling efficient conversion of CO and H_2_ at high pressures and temperatures. Its high activity and stability under industrial conditions made large-scale methanol production viable. Initially designed for CO hydrogenation, this catalyst also laid the groundwork for its subsequent application in CO_2_ hydrogenation, as Zn-based catalysts were later adapted to facilitate methanol synthesis from CO_2_-rich feedstocks, demonstrating their versatility in both CO and CO_2_ conversion pathways.

ZnO–ZrO_2_ (ZZO) catalysts are notable for their thermal stability, which is crucial for high-temperature catalytic processes like methanol synthesis. Their resistance to sintering makes them valuable for long-term use in industrial applications. ZnO–ZrO_2_ solid solution catalyst reported by Wang *et al.* exhibited high methanol selectivity of 86–91% with CO_2_ conversion over 10% under industrial conditions.^89^ The catalyst showed stability for over 500 hours and resistance to sulfur-containing compounds, a common issue in CO_2_ sources. High performance was attributed to the synergetic effect between Zn and Zr sites, enhancing the activation of both H_2_ and CO_2_. Authors also observed a significant performance variation based on the Zn/(Zn + Zr) molar ratio with the optimized performance when 13% ZnO–ZrO_2_ catalyst was employed. Another synthesis method was employed by Pinheiro Araújo *et al.* Flame spray pyrolysis method enhanced surface area of ZnZrO_*x*_ catalyst and promoted the formation of atomically dispersed Zn^2+^ sites, which are crucial for improved methanol productivity. Threefold higher methanol productivity compared to their coprecipitation counterparts was reported due to the maximized surface area and the formation of active ensembles that include oxygen vacancies and neighbouring Zn and Zr atoms, which favour methanol formation while suppressing undesired CO production.^90^ ZnZrO catalyst was also promoted with indium. In the system developed by Zhou *et al.* 2.5 wt% In_2_O_3_ was added to ZZO. This combination led to a significant improvement in created surface oxygen vacancies that, in turn, facilitated CO_2_ adsorption and hydrogen activation, aiding to achieve CO_2_ conversion rate of 13.5% and a methanol space-time yield of 0.749 g g_cat_^−1^ h^−1^ at 330 °C. The catalyst maintained typical ZZO stability showing no decline in the performance for over 200 hours of operation.^91^

#### Production of aromatics from methanol

Aromatics, such as benzene, toluene, and xylene, are crucial for the chemical industry and essential components of SAFs between 14–19%. The Methanol-to-Aromatics (MTA) process is particularly significant for producing these valuable compounds. This section details the MTA process with a particular focus on catalyst development, which is critical for enhancing efficiency and selectivity in the production of aromatics from methanol.^78,98^

The MTA process involves the conversion of methanol into a mixture of aromatic hydrocarbons, primarily benzene, toluene, and xylene (BTX), along with minor amounts of other C_6_–C_12_ hydrocarbons. This conversion is typically carried out using zeolite-based catalysts, which provide the necessary acidic sites to facilitate the transformation reactions. The process is of great interest due to the increasing demand for aromatics and the need to find alternative routes to produce these compounds as petroleum resources deplete.

The production of aromatics from methanol involves several essential stages. Initially, methanol converts to olefins by forming the first C–C bond, facilitated by surface methoxy species (SMS) reacting to create hydrocarbons. These hydrocarbons then engage in a “hydrocarbon pool” mechanism, where olefins and aromatics interconvert, promoting continuous olefin formation. Subsequently, metal-modified ZSM-5 catalysts, such as those containing Zn or Ga, provide Lewis acid sites that assist in the dehydrogenation of olefins to dienes. These dienes, crucial intermediates, undergo cyclization to form aromatic rings. The cyclization and stabilization of these aromatic rings require the synergistic action of Brønsted acid sites from the ZSM-5 structure and Lewis acid sites introduced through metal modifications. The final step involves stabilizing and forming benzene, toluene, and xylene (BTX), influenced by specific reaction conditions and the nature of the catalyst.^99–101^

Incorporating metals like Zn and Ga into zeolites, especially ZSM-5, enhances the dehydrogenation capability of the methanol-to-aromatics (MTA) catalyst, thereby improving the selectivity for aromatic compounds. These metal species can be integrated into zeolites through various methods, including mechanical mixing with metal oxides, direct hydrothermal synthesis, ion exchange, incipient wet impregnation, chemical vapor deposition (CVD), and atomic layer deposition (ALD). The chosen preparation method significantly impacts the structural features of the metal sites, such as their nature, loading, and stability.

Gallium (Ga) and aluminium (Al) are very similar and can be incorporated into the zeolite structure, creating bridging hydroxyl groups. During the synthesis of zeolites with MFI topology, Ga is added to the precursor solution and subjected to hydrothermal treatment. The substitution of Al by Ga decreases the acidic strength, and post-thermal treatments allow Ga to migrate to extra-framework positions, resulting in well-dispersed Ga species that effectively interact with Brønsted acid sites. For example, nanostructured [Al]-, [Ga, Al]- and [Ga]-HZSM-5 catalysts prepared by direct hydrothermal synthesis showed that a [Ga, Al] HZSM-5 catalyst with a Ga/Al ratio of 0.5 and a low Ga/Brønsted acid ratio of 0.06 exhibited the highest aromatic selectivity^102,103^ (see Table 3, entry 1).

**Table 3:** Experimental details and catalytic performance for methanol-to-aromatics conversion of all the catalysts mentioned in this manuscript

Entry	Catalyst	Temp. (°C)	Pressure (MPa)	Space velocity (mL g^−1^ h^−1^)	Product distribution (%)			Life time^a^	Ref.
					C_1_–C_4_	C_5+_	BTX		
1	Ga-Al-MFI	390	0.5	4	50	27	33	80 h (50%)	102
2	Zn-HZSM-5	400	0.1	2.5	32.4	11.9	55.3	120 h (90%)	104
3	Cd-ZSM-5	420	0.1	2.1	25.2	11.8	63	4 h (20%)	105
4	Zn-ZSM-5	420	0.1	2.1	32.5	10.9	56.6	4 h (20%)	105
5	La-HZMS-5	437	0.1	1	35.6	7	50	40 h (17.5%)	106
6	Zn-nanoHZSM-5	475	0.1	1	n.r.^b^	n.r.	70	300 h (100%)	107
7	Zn-NZS	475	0.1	1	24.35	7.9	67.9	70 h (50%)	108
8	Zn-CZS	475	0.1	1	16.77	31.23	52	2.8 h (50%)	108
9	Ga-CN-HZSM-5	500	0.1	5	22.7	4.7	72.6	12 h (30%)	109
10	Ga-HZSM-5	500	0.1	5	45.6	10.4	44	15 h (30%)	109
11	Ga/HDZSM-5	500	0.1	2.5	34.8	7.5	57.7	14 h (30%)	110
12	Ga/HZSM-5	500	0.1	2.5	39.4	9.5	50.7	12 h (30%)	110
13	NZ5	400	0.1	20	n.r.	n.r.	50	16 h (35%)	111
14	DeSi	400	0.1	20	n.r.	n.r.	70	20 h (75%)	111

a *X* h (*Y*%) stands for a reaction lasting for *X* hours before the methanol conversion decreases to *Y*%.

b n.r. – not reported.

Incorporating zinc (Zn) into the MFI framework presents more challenges, as Zn tends to migrate to extra-framework positions upon heating. Direct synthesis of Zn-ZSM-5 results in well-dispersed extra-framework Zn species, although it may affect zeolite crystallization, leading to smaller crystallite sizes. Ion exchange can introduce Zn into zeolites, forming well-defined Zn^2+^ species. Techniques such as IR spectroscopy, temperature-programmed desorption, X-ray absorption spectroscopy, and ^27^Al NMR spectroscopy have provided insights into the configurations of Zn^2+^ species, including isolated Zn^2+^ cations and oxygen-bridged Zn^2+^ ion pairs. ZnOH^+^ species, stabilized in the ZSM-5 framework, are particularly active for aromatization, especially in catalysts with high Si/Al ratios^104,112^ (Table 3, entry 2).

Other metals such as Ag,^113,114^ Cd,^105^ Mo,^115^ and La^106^ have also been tested in H-ZSM-5 catalysts for MTA. Ag-ZSM-5 demonstrates activity but can be reduced to inactive metallic Ag. Cd-modified H-ZSM-5 exhibits better performance compared to Zn-ZSM-5, though its toxicity poses a limitation for its use. The inclusion of Mo enhances aromatic selectivity, but it results in catalyst deactivation due to sintering. Incorporating La, on the other hand, extends the catalyst's lifespan and boosts the yield of BTX (see Table 3, entries 3–5).

The concentration and strength of acid sites play a crucial role in determining product selectivity in the methanol-to-aromatics (MTA) process. Typically, an increase in Brønsted acid sites enhances hydride transfer and cyclization reactions, which in turn promotes the formation of aromatics. The introduction of Zn or Ga into zeolites can further alter their acidity. Wang *et al.*^116^ studied the variations in acidic properties of H-ZSM-5 and Zn-ZSM-5. The NH_3_-TPD profiles enable the quantification of the number of strong, medium, and weak acid sites by measuring ammonia desorption at different temperatures (300–550 °C for strong sites, 200–300 °C for medium sites, and 120–200 °C for weak sites). In metal-modified catalysts, such as Zn-ZSM-5, an increase in the number of medium-strength acid sites is observed. The introduction of metals like Zn and Ga into the zeolite structure not only affects the quantity but also the distribution of acid sites. These metals introduce additional Lewis acid sites, in addition to the Brønsted acid sites provided by the zeolite structure.

The deactivation of zeolites by coke deposition is one of the most limiting factors in this process. In order to generate a large number of aromatics before catalyst regeneration, it is essential to improve the catalysts' resistance to coke formation. Reducing the aluminium content in zeolites or introducing rare earth metals can alter the acidic properties of zeolite-based catalysts, thus slowing down coke formation. In the case of the ZSM-5 zeolite, its purely microporous channels provide optimal shape selectivity to produce BTX products, but also result in slow diffusion of reagents and bulky products or coke precursors that cause them to re-react and form unwanted products. To address diffusion limitations, the use of zeolites with enhanced diffusion properties is a viable alternative approach.^117–121^

Reducing the crystal size of zeolites is an effective method to enhance molecular transport by shortening diffusion pathways. The synthesis of nanosized zeolite crystals, particularly those with at least one dimension under 100 nm, has been a key area of focus in zeolite research. Approaches to achieve this are generally categorized into conventional and nonconventional synthesis methods.

In conventional synthesis, systems with high nucleation rates tend to produce smaller crystallites. Various factors influence the crystal size of ZSM-5 zeolites, including the type and amount of template molecules, the aging process, temperature, crystallization time, and water content. For example, Wang*et al.*^122^ found that increasing the amount of colloid silicalite-1 seeds in the synthesis gel reduced the crystal size of ZSM-5 zeolites. These resulting crystals, ranging in size from 0.25 μm to 2 μm, showed different distributions of Zn species (ZnOH^+^ and ZnO) after zinc doping. The smallest Zn-ZSM-5 catalysts, with a higher proportion of ZnOH^+^ species, demonstrated the highest aromatic selectivity and the longest lifespan in the MTA reaction.

Moreover, Qian *et al.*^107^ used urea as an additive to restrict the growth of ZSM-5 crystals along the *b*-axis, synthesizing nanosized ZSM-5 with a *b*-axis around 60 nm. High-resolution techniques like high-angle annular dark-field scanning transmission electron microscopy (HR HAADF-STEM) and integrated differential phase contrast (iDPC) imaging showed that all straight channels were fully opened. This facilitated the rapid diffusion of aromatic products from the (010) crystal plane, achieving nearly 100% methanol conversion on Zn-modified nano-ZSM-5 catalysts over approximately 300 hours at 475 °C (see Table 3, entry 6). However, it is important to note that about 70% of the aromatic product consisted of trimethylbenzenes and heavier multimethylbenzene, with BTX selectivity being less than 30%.

Nonconventional methods for synthesizing nanosized zeolite crystals often involve strategies to limit Ostwald ripening, typically by using sacrificial templates to constrain the space for crystal growth. For example, porous carbon can serve as an inert template where the zeolite precursor gel is confined within the pores, allowing for the formation of nanosized zeolite crystals after the template is removed.^123,124^

The dry gel conversion (DGC) method is another innovative approach that has gained popularity. This method enhances the solid yield of zeolites and reduces waste.^125^ In DGC, the solid components (like zeolite precursor powder) are placed in a perforated Teflon basket above a liquid at the bottom of a liner. As the liquid heats and vaporizes, steam facilitates the crystallization of the zeolites. Qian *et al.*^108^ utilized this technique to produce nanosized ZSM-5 crystals with consistent sizes and adjustable Si/Al molar ratios. One notable result of using the DGC method was the creation of nanosized Zn-ZSM-5 with a Si/Al ratio of 60 (Zn/NZS-60), which showed a catalytic lifespan nearly 25 times longer than its conventional micrometer-sized counterpart (Zn/CZS-60). Examination of the used catalysts indicated that the nanosized crystals had a greater capacity to retain coke and exhibited a slower coke formation rate. Importantly, the selectivity towards 1,2,4-trimethylbenzene and heavier aromatics was 67%, compared to the micrometer-sized Zn-ZSM-5, where BTX constituted the majority (78%) of the aromatic hydrocarbons. This variance is likely due to the larger external surface area of nanosized zeolites, which facilitates the conversion of xylene into heavier compounds^108^ (see Table 3, entries 7 and 8).

Hierarchically structured zeolites, which combine intracrystalline mesopores with the zeolite's intrinsic micropores, are highly effective in extending the lifespan of catalysts.^126,127^

Lin *et al.*^109^ recently created a hierarchical Ga-CNT-HZSM-5 catalyst by using Ga-immobilized carbon nanotubes as hard templates (bottom-up approach). The well-dispersed Ga species and the abundant mesopores in this catalyst led to improved aromatics yield and increased resistance to deactivation *versus* the Ga-HZSM-5 (Table 3, entries 9 and 10).

Various Ga-HZSM-5 catalysts were synthesized and tested for methanol aromatization at high temperatures (400–500 °C) by Po-Chen Lai *et al.*^110^ Desilication (top-down approach) led to mesopore expansion and improved Lewis and Brønsted acidities, which facilitated better mass transfer and enhanced the migration of gallium ions into the zeolite structure. This resulted in the formation of bifunctional sites (GaO)^+^-Brønsted acid centers, promoting aromatics production through dehydrogenation and cyclization processes. The study found that Ga-doped desilicated HZSM-5 (Ga/HDZSM-5) catalysts exhibited higher aromatics yield and increased resistance to deactivation by coking, compared to untreated ZSM-5 (Ga/HZSM-5) (see Table 3, entries 11 and 12).

Similary, Liu *et al.*^111^ investigate the impact of desilication on ZSM-5 (NZ5) zeolites for methanol-to-aromatics (MTA) reactions. By using tetrapropylammonium hydroxide (TPAOH) for post-synthetic desilication, the study explores variations in mesoporosity and acidity, assessing their effects on catalyst stability and coke formation. The desilicated ZSM-5 zeolites (DeSi) showed increased mesoporosity and decreased strong acid sites, which helped in reducing coke formation and improving catalytic stability. NH_3_-TPD and FT-IR analyses revealed that the treatment with TPAOH enhanced mesoporosity while slightly reducing strong Brønsted acid sites, crucial for aromatization. The study concludes that moderate alkali treatment (*e.g.*, DeSi) results in a notable decrease in Brønsted acid sites, improving catalytic performance by balancing acidity and mesoporosity, thereby enhancing the stability and selectivity for aromatic compounds in the MTA process (see Table 3, entries 13 and 14).

#### Production of olefins from methanol

The production of olefins, which can subsequently be oligomerized into hydrocarbons suitable for jet fuel, is crucial for the effective utilization of CO_2_ as a raw feedstock. One of the most promising strategies involves using methanol derived from CO_2_ and converting it into olefins. In this section, we will explore the leading catalysts for this transformation and the methods developed to enhance their selectivity and, importantly, their longevity.

Methanol-to-olefins (MTO) conversion is a significant reaction in C1 chemistry, offering a pathway to produce essential petrochemicals, like ethylene and propylene, from non-petroleum sources such as CO_2_. Since its inception in the 1970s, the MTO process has evolved, with commercial applications realized in 2010. The key to its success lies in the development of efficient catalysts, particularly shape-selective catalysts like ZSM-5 and SAPO-34, which enable high methanol conversion rates with high selectivity towards light olefins.^128^

The MTO process begins with the conversion of methanol to dimethyl ether (DME), followed by the transformation of methanol and DME to olefins over solid acid catalysts. While amorphous solid acids like Al_2_O_3_ and Al_2_O_3_–SiO_2_ can facilitate methanol to DME conversion, they lack high selectivity for light olefins. The introduction of molecular sieve catalysts, notably ZSM-5 and SAPO-34, revolutionized the process, significantly enhancing the selectivity for light olefins while minimizing the production of undesired higher hydrocarbons.^129^

As will be discussed in the subsequent sections, the oligomerization process to produce longer chain hydrocarbons for aviation fuel varies depending on whether short or long olefins are used as the starting material. This process requires different catalysts and reaction conditions. Additionally, these olefins are crucial for plastics production. Consequently, significant efforts have been dedicated to developing catalysts that are selective for a specific range of olefins.

ZSM-5 is known for its strong acidity and high thermal stability, making it an excellent catalyst for the MTO process. Its structure facilitates the formation of hydrocarbons through a mechanism involving the surface methoxy species (SMS) and the hydrocarbon pool (HCP).^76^ The HCP mechanism allows for the continuous generation of olefins by cyclic hydrocarbons acting as intermediates. However, the strong acidity of ZSM-5 can also lead to rapid catalyst deactivation due to coke formation. Research has focused on modifying the acidity of ZSM-5, either by incorporating metals or by adjusting the Si/Al ratio, to enhance its catalytic performance and longevity.^130^

Another approach to extending the lifetime of these catalysts involves introducing mesoporosity into the structure. This modification allows for faster diffusion of the produced compounds, preventing them from undergoing further reactions that lead to coke formation or from blocking the pores. The preparation of mesoporous zeolites is an extensive and well-researched area, deserving of its own detailed review. Numerous studies on this topic are available, and we highly recommend readers to explore these works for a deeper.^131–140^

The first method is the bottom-up approach, which involves adding a “porogen” during the zeolite synthesis. This porogen is later removed through leaching or thermal treatment. An example of this is published by Feng *et al.*^141^ They investigate the enhancement of ZSM-5 catalysts for methanol to propylene (MTP) reactions through a two-stage glucose-assisted crystallization method. This approach yielded hierarchical ZSM-5 zeolites with large prismatic crystallites and increased intracrystalline mesopores, while also reducing acid strength and total acid site density. Structural characterization techniques, including XRD, FTIR, and NH_3_-TPD, confirmed these improvements. Catalytic testing revealed that the newly prepared ZSM-5-T3 catalyst exhibited a propylene selectivity of 45.2% and a high propylene to ethylene ratio of 8.4. The enhanced mesoporosity and moderated acidity contributed to improved product diffusion and reduced coke formation, leading to longer catalyst lifetimes and higher propylene yields compared to traditional one-stage crystallized ZSM-5-T0 catalysts (Table 4, entries 1 and 2).

**Table 4:** Experimental details and catalytic performance for methanol-to-hydrocarbon conversion of all the catalysts mentioned in this manuscript

Entry	Catalyst	Temp. (°C)	Pressure (MPa)	Space velocity (mL g^−1^ h^−1^)	Product distribution (%)			Life time^a^	Ref.
					C_1_–C_4_	C^=^_2_–C^=^_4_	Others		
1	ZSM-5-T3	450	0.1	4	0.4	63.9	35.6	9 h (85%)	141
2	ZSM-5-T0	450	0.1	4	1.8	29.1	69.1	6 h (85%)	141
3	MHZ5	400	1	10	n.r.^b^	n.r	35^c^	145 h (65%)	142
4	Z50-H1	350	0.1	9.5	15	40	45	50 h (50%)	143
5	Z50-C	350	0.1	9.5	16	46	38	26 h (50%)	143
6	nt-SAPO-34	470	0.1	1	2	80	18	6 h (50%)	144
7	p-SAPO-34	470	0.1	1	3	79	18	1.5 h (50%)	144
8	dsi-SAPO-34	400	0.1	0.73	2	80	18	10 h (50%)	145
9	un-SAPO-34	400	0.1	0.73	1.8	80.8	18	5 h (50%)	145

a *X* h (*Y*%) stands for a reaction lasting for *X* hours before the methanol conversion decreases to *Y*%.

b n.r. – not reported.

c Liquid hydrocarbon.

The second method is the top-down approach (see Fig. 5), where purely microporous zeolites are first synthesized, and then mesopores or macropores are introduced through post-synthesis treatments. This is typically achieved by partially removing aluminum (dealumination) and/or silicon (desilication) atoms from the zeolite framework. Techniques such as steaming or acid treatment are commonly used to create mesopores in this manner. However, it's important to note that dealumination can result in a loss of acid sites, which may negatively impact reactions catalyzed by Brønsted acid sites.^123,125^

Fu *et al.*^142^ developed a series of nanosized ZSM-5 zeolites with different pore structures to enhance their catalytic performance for methanol-to-hydrocarbons (MTH) reactions. By employing a controllable desilication process using NaOH and tetrapropylammonium hydroxide (TPAOH), they successfully created mesoporous and hollow structures within the ZSM-5 crystals. The research demonstrated that these modifications significantly improved the catalysts' diffusion properties, allowing better access to active sites and reducing coke formation, which typically deactivates catalysts. Specifically, the hollow ZSM-5 with rich mesopores in the shell (MH-Z5) (Table 4, entry 3) exhibited the highest catalytic lifetime of 149 hours, attributed to its enhanced mesoporosity and improved diffusion characteristics.

Pérez-Ramírez *et al.*^143^ employed a top-down demetallation strategy to introduce mesopores in specific regions of MFI-type zeolite crystals, maintaining identical bulk porous and acidic properties. They compared this method with bottom-up approaches like carbon templating and seed silanization. Advanced characterization techniques, including positron annihilation lifetime spectroscopy (PALS), were used to evaluate mesopore size, distribution, and connectivity. The findings revealed that mesopore quality, defined by the connectivity and accessibility of the mesopores, is crucial for enhancing the catalytic performance and prolonging the catalyst's lifetime. Zeolites with well-connected and open mesopores (Z50-H1) demonstrated superior resistance to coking and extended operational lifetimes in methanol-to-hydrocarbons (MTH) compared to those with constricted or poorly connected mesopores (Z50-C) see (Table 4, entries 4 and 5).

SAPO-34, on the other hand, offers a different set of advantages. It has a smaller pore size, which restricts the formation of larger hydrocarbons and thus enhances the selectivity towards light olefins like ethylene and propylene. SAPO-34 also exhibits a high degree of hydrothermal stability and can maintain its catalytic activity over extended periods. The main challenge with SAPO-34 is its susceptibility to deactivation due to the formation of coke within its pores. To address this, researchers have explored various strategies such as the addition of promoters, the use of hierarchical structures, and the incorporation of secondary mesoporosity to improve the diffusion of reactants and products, thereby reducing coke deposition.

Like the ZSM-5, the SAPO-34 can be mesostructured to improve resistance to deactivation as well. An example of a bottom-up is the one that was published by Kaskel *et al.*^144^ that focus on the development of carbon-templated SAPO-34 catalysts with enhanced adsorption kinetics and catalytic performance for methanol-to-olefins (MTO) reactions. The researchers introduced transport pores into crystalline SAPO-34 using carbon nanoparticles and nanotubes as hard templates during hydrothermal synthesis. Characterization techniques, including XRD, SEM, NMR, NH_3_-TPD, EDX, and N_2_-physisorption, confirmed the presence and accessibility of mesopores. The carbon nanotube-templated SAPO-34 (nt-SAPO-34) showed a significant improvement in *n*-butane uptake and methanol conversion with respect to a parent p-SAPO-34, attributed to mesopores accessible from the particle's exterior, enhancing diffusion and catalytic performance. These mesopores remained stable after multiple regeneration cycles, indicating their potential for long-term catalytic applications in MTO processes (Table 4, entries 6 and 7).

An example of a top-down synthesis of mesoporous SAPO-37 is published by Liu *et al.*^145^ They developed a facile tetraethylammonium hydroxide (TEAOH) etching post-treatment method to create hierarchical SAPO-34 (dsi-SAPO-34) crystals with enhanced pore structures. This treatment introduced meso- and macropores within the zeolite, significantly improving mass transport and diffusion efficiency. As a result, the single-run lifetime of the treated SAPO-34 (dsi-SAPO-34) catalyst in the MTO process was doubled from 320 to 640 minutes with the un treated (un-SAPO-34) maintaining high methanol conversion and selectivity towards light olefins. The dsi-SAPO-34 also exhibited high stability during reactivation cycling, indicating its potential for industrial applications.

The evolution of mesoporous zeolites for MTO catalysis represents a significant step forward in the quest for efficient and durable catalysts. By addressing the challenges of diffusion limitations and coke formation, these materials pave the way for more sustainable and high-performing catalytic processes.

#### Olefin oligomerization

Ethylene, propylene, and butylenes, common products of MTO and high-temperature FTS, can be subsequently transformed into higher hydrocarbons through oligomerization, which is a key step towards producing jet fuel range.

#### Oligomerization of light olefins

Traditionally, olefins production hinges on the steam thermal cracking of hydrocarbons, a staple process in the petrochemical industry. Yet, innovative approaches that leverage alternative feedstocks such as natural gas, coal, biomass or CO_2_ are gaining industrial traction. Notably, the conversion of methanol into olefins through a zeolite-catalyzed methanol-to-olefin (MTO) process, as well as the transformation of bio-ethanol into olefines, are emerging as highly promising methodologies.

The process of oligomerizing light olefins in many cases, including ethylene, stands as a vital pathway to synthesize linear and branched higher olefins and paraffins. These compounds have diverse applications, ranging from the creation of detergents and petrochemicals to serving as oil additives and components in high-octane eco-friendly gasoline. Specifically, for jet fuel production, catalytic olefins oligomerization to create C_8+_ hydrocarbons might be of paramount importance.^146^

While the oligomerization of ethylene has been traditionally mediated by homogeneous and heterogeneous catalysts, the industrial production of these higher olefins often employs homogeneous acidic catalysts. For example, the Shell Higher Olefin Process (SHOP)^147,148^ utilizes nickel complexes to produce a spectrum of higher olefins from C_4_ to C_20+_, and the Sabic/Linde Alpha-SABLIN process harnesses zirconium with an alkyl aluminum co-catalyst.^149^ Over recent decades, a variety of new and potent homogeneous catalysts including complexes of nickel, chromium, zirconium, tantalum, titanium, hafnium, cobalt, iron, and tungsten have been developed, enhancing the process efficiency.^150–153^ Acid catalysts are used for the oligomerisation of C_3_ or longer olefins *e.g.* supported phosphoric acid (Catpoly process)^154^ and zeolites (Mobil olefins to gasoline and distillates (MOGD) process).^155^ Olefins produced through MTO are generally a mixture ranging from C^=^_2_ to C^=^_4_ so the catalysts used must have a multifunctional character in which they are able to oligomerise different olefins in one reactor under the same conditions.

For this reason, the scientific community has been working on the fabrication of heterogeneous catalysts by anchoring organometallic complexes on supports such as silica and MCM-41, although these systems often fall short in activity and stability compared to their homogeneous equivalents. A diverse array of Ni-containing porous materials, including Ni-exchanged amorphous silica-alumina,^156–158^ Ni/sulfated alumina,^159^ Ni-zeolites,^160–162^ Ni-AlMCM-41,^159,161^ and Ni-AlSBA-15,^163^ have demonstrated their efficacy as catalysts for the oligomerization of olefin. Ni^2+^ catalysts excel in facilitating the dimerization or trimerization producing short-chain olefins. While these resulting olefins are not directly applicable for the production of jet fuel, they can undergo further polymerization to produce longer hydrocarbon chains, which are in the range of jet fuel requirements.^164^

These initial products may further polymerize due to the acidic aspect of the support. Specifically, acidic Brønsted sites present in solid acid catalysts, like zeolites, can protonate olefins, resulting in the creation of secondary or tertiary carbocations. These ions are then involved in chain growth reactions, contributing to the synthesis of larger hydrocarbon molecules.^165^ Within these bifunctional catalysts,^166^ the primary reaction mechanism unfolds at the Ni^2+^-acid sites, predominantly *via* the Cosse-Arlman mechanism but not exclusively.^167,168^ Nonetheless, the acid sites independently can also serve as catalysts for oligomerization at higher temperatures. Moreover, zeolites facilitate various parallel reactions, including isomerization (may be beneficial), hydrogen transfer, cracking, cyclization, and coking, although not all these processes yield desired outcomes.^169^

Moreover, the effectiveness of these Ni-based catalysts, specifically in terms conversion, hinges on the availability of Ni sites. Meanwhile, factors such as the stability of the catalyst, the distribution of carbon numbers, and the ultimate structure of the oligomers are influenced by the density and strength of the Brønsted–Lewis acid sites, as well as the porosity of the catalyst.

For instance, zeolites that have undergone nickel exchange (such as Ni–Y and Ni-MCM-22) experience rapid deactivation as their micropores become blocked by heavier oligomer accumulations.^170,171^ Lallemand *et al.*^172^ observed that the MCM-36 zeolite, once exchanged with Ni, demonstrated superior activity and stability compared to its MCM-22 counterpart. This enhancement is attributed to the mesoporous structure of MCM-36, which aids in the diffusion of larger oligomers produced throughout the reaction process (see Table 5, entries 1 and 2). Mohamed *et al.*^178^ demonstrated that the use of hierarchically structured zeolites enhances catalyst stability by facilitating the removal of coke precursors from the zeolite structure more effectively than with smaller pores.

**Table 5:** Experimental details and catalytic performance for olefin oligomerization of the catalysts mentioned in this manuscript

Entry	Catalyst	Temp. (°C)	Substrate	Pressure (MPa)	Type of reactor	Space velocity (h^−1^)	Product	Ref.
1	Ni-MCM-22	150	Ethylene	4	Batch	n.a.	2.5 g of oligomers per g of catalyst	172
2	Ni-MCM-36	150	Ethylene	4	Batch	n.a.	46 g of oligomers per g of catalysts	172
3	Ni-AlMCM-41	150	Ethylene	3.5	Batch	n.a.	175 g of oligomers per g of catalysts	173
4	Ni-Siral-30	200	Ethylene	1	Flow reactor	0.375	Conversion 70%, selectivity towards C_10 +_ 20%	174
5	Ni-meso-H-Ni-ZSM-5	200	Ethylene	3.5	Flow reactor	2.5	Conversion 40%, selectivity towards C_10+_ 90%	175
6	Ni-meso-H-beta	200	Ethylene	3.5	Flow reactor	2.5	Conversion 50% selectivity towards C_10+_ 80%	175
7	Ni-H-ZSM-5	200	Ethylene	3.5	Flow reactor	2.5	Conversion 20%, selectivity towards C_10+_ 10%	175
8	Ni-H-beta	200	Ethylene	3.5	Flow reactor	2.5	Conversion 35%, selectivity towards C_10+_ 10%	175
9	SZS-5	220	1-Hexene	4	Flow reactor	1	Conversion 38%, selectivity towards C_10+_ 40%	176
10	dSZS-5	220	1-Hexene	4	Flow reactor	1	Conversion 90%, selectivity towards C_10+_ 55%	176
11	Ni-AlKIT-6	240	Ethylene	2	Flow reactor	1	Conversion 95.9, selectivity towards C_10+_ 56.5%	177

Aluminosilicates exchanged with Ni that possess larger pores, such as Ni–SiO_2_–Al_2_O_3_,^179^ Ni-AlMCM-41,^170–173,180^ and Ni-AlMCM-48, have been identified as highly active and less prone to deactivation. More recently, Andrei *et al.*^173^ highlighted that Ni-exchanged AlSBA-15 catalysts stand out for their exceptional performance in the oligomerization of olefins, offering enhanced activity and stability both in batch and flow reactors^163^ (see Table 5, entry 3). This efficiency is attributed to their interconnected mesopores, which are sufficiently large to facilitate the passage of heavier oligomers, thus reducing the rate of deactivation.

Literature reviews indicate that most catalytic systems favour the production of hydrocarbons following a Schulz–Flory type distribution, with a preference for shorter chains (*i.e.*, C_4_ > C_6_ > C_8_ > C_10+_). However, generating hydrocarbons with more than ten carbon atoms, essential for the creation of high-quality jet or diesel fuels, presents a significant challenge.^181,182^

Despite these limitations in achieving long hydrocarbon chains, there have been attempts to generate jet fuel catalysts in a single step by oligomerisation either of ethene or propene. Thus, Hwang *et al.*^174^ used as Ni supported on a material developed by Sasol called Siralox-30, which is a material with medium acidity and high mesoporosity. They used it for oligomerisation of ethylene and found that oligomers in the Jet-Fuel range can be generated. However, the degree of deactivation was high (Table 5, entry 4).

Moon *et al.*^175^ explored the oligomerization of propene process over NiH- and H-forms of ZSM-5 and beta zeolite catalysts, highlighting the significance of modifying textural properties to enhance the efficiency and stability of the catalysts for producing liquid fuel range products, particularly those beyond C_10+_. This paper emphasizes the advantage of employing zeolites with modified textural properties, such as increased mesoporosity (Ni-meso-H-ZSM-5 and Ni-meso-H-beta), to overcome diffusion limitations commonly encountered in the oligomerization process and allowing heavy hydrocarbons to diffuse out the zeolite. The study demonstrates that nanometer-scale, sheet-like crystals along with intercrystalline mesoporosity significantly contribute to the higher efficiency of the oligomerization process. Specifically, the researchers show that the Ni-meso-H-ZSM-5 and Ni-meso-H-beta zeolites, modified to include nanocrystalline and mesoporous structures, exhibit improved catalytic performance compared to their counterparts without such modifications. The conversion drops over time, which shows that the catalyst is deactivated.

Monama *et al.*^176^ published a work, the focus was on enhancing the textural properties of ZSM-5 (called SZS-5) catalysts through desilication to boost their performance in oligomerizing light olefins such as propylene and 1-hexene into fuel-range hydrocarbons, particularly emphasizing jet fuel generation. Mesoporous ZSM-5 (dSZS-5), created *via* post-synthesis alkaline treatment, exhibited an exceptional increase in activity and selectivity towards jet fuel/diesel range products, reaching about 90% conversion and 55% selectivity under optimal conditions. The introduction of mesoporosity was confirmed to not compromise the microporous structure, preserving the catalyst's crystallinity and maintaining its acidic sites, essential for the oligomerization process. The desilicated catalysts were active for more than 80 hours (see Table 5, entries 9 and 10).

Panpian *et al.*^177^ synthesized KIT-6 (Korean Institute of Technology) using a sol–gel method, followed by post-synthetic alumination, with nickel being incorporated *via* incipient wetness impregnation. The performance of NiAlKIT-6 was meticulously evaluated in a continuous fixed-bed reactor, examining its catalytic activity across varying conditions. A notable observation was that higher Si : Al ratios and nickel loadings significantly bolstered ethylene conversion and C_8+_ selectivity. However, excessive alumination altered the pore structure, adversely affecting the catalyst's activity and selectivity (see Table 5 entry 11).

The catalyst showcased a good ethylene conversion rate exceeding 95%, with C_8+_ selectivity reaching up to 55%. Its stability remained robust over a 30 hours time on stream, maintaining high selectivity with a mild degradation. Additionally, the spent catalyst could be regenerated, preserving its catalytic activity effectively.

This research emphasizes the critical role of mesoporosity in enhancing catalyst performance for ethylene oligomerization towards jet fuel production. The tailored mesoporous structure of NiAlKIT-6 facilitated the diffusion of larger oligomers, significantly minimizing catalyst deactivation. The study successfully demonstrates the potential of optimized NiAlKIT-6 catalysts in the sustainable production of bio-jet fuel.

#### Two step oligomerization

These instances underscore the necessity of bifunctional Ni–acid catalysts for the oligomerization of short-chain olefins, particularly ethylene, to synthesize hydrocarbons within the jet fuel range. In these endeavors, Ni is supported on acidic catalysts like zeolites, creating bifunctional materials that facilitate the reaction process. An innovative alternative involves employing two catalysts, offering enhanced versatility. With this approach, one catalyst, comprised of a supported Ni catalyst (regardless of whether the support itself is acidic), targets the dimerization or trimerization of shorter-chain olefins like ethylene. The resultant compounds then can be put in contact with the second catalyst, where an acid catalyst promotes the oligomerization of these longer-chain hydrocarbons.

The dual approach effectively distinguishes the preparation processes of Ni and acid catalysts, thereby broadening the synthesis and preparation flexibility of both types of catalysts. For instance, sulphonated macroporous resins like Amberlyst-30 are recognized for their efficacy in oligomerizing butenes.^183^ Nevertheless, their polymeric composition challenges their application as metal supports, primarily because they cannot withstand heat treatments necessary for decomposing metal precursors. When Ni is anchored on zeolites or acidic inorganic substrates, it often alters the acidity of the zeolite, occasionally resulting in a final acidity level that is not optimal. Crafting these bifunctional catalysts demands meticulous effort, and the use of two catalysts enhances our ability to finely tune the properties of the catalysts to achieve the desired reaction outcomes efficiently.

#### Batch processes

Hulea *et al.*^173^ report this approach for the first time. They conducted a two-stage oligomerization process using a batch reactor, where ethylene was first converted into liquid products through a catalyst (Ni supported on MCM-41). In the subsequent stage, the olefins produced were further oligomerized using an Al-exchanged MCM-41 catalyst (consecutive oligomerization). This sequential oligomerization led to a chain growth of the produced olefins, although it did not achieve the molecular length required for jet fuels.

Additionally, an experiment was conducted with both catalysts simultaneously present in the reactor. This approach yielded hydrocarbons within the jet fuel range, indicating a successful production method. It is important to note that the two methods described were performed under slightly different conditions so the results are not very comparable. However, it is a good proof of concept that the combination in the same reactor of catalysts suitable for ethylene oligomerisation and another one specialised in heavier olefines oligomerisation are compatible and work cooperatively.^73^

The outcomes derived from two separate methodologies conclusively demonstrated that the oligomers formed in the initial phase with nickel-exchanged catalysts were capable of undergoing subsequent reactions on the acidic catalyst, culminating in the production of heavier oligomers. Furthermore, it became clear that each catalyst was responsible for facilitating distinct chemical transformations, proceeding through two mechanistically different routes. Initially, nickel ions functioned as active sites for ethylene's dimerization into 1-butene, followed by further oligomerization reactions that involved combinations such as 1-butene–ethylene, 1-hexene–ethylene, among others (Fig. 5).

Babu and colleagues^184^ adopted a two-step method for producing jet fuel from ethylene. Initially, they utilized a Ni-AlSBA-15 catalyst to oligomerize ethylene into higher olefins, which were then further polymerized into jet fuel range hydrocarbons using Amberlyst-35 as the catalyst. The oligomerization occurred at 200 °C and 10 bar, while the subsequent polymerization took place at 100 °C and 30 bar. The necessity for differing conditions—stemming from Amberlyst-35's instability at temperatures above 150 °C—mandated the bifurcated process. The authors noted the requirement to wash Amberlyst-35 with *n*-heptane post-reaction to remove strongly adsorbed hydrocarbons, enabling its reuse.

This dual-stage methodology allows for the precise tuning of reaction environments to each catalyst's needs, presenting a compelling approach for laboratory investigations. However, this strategy introduces significant complexity at a practical and industrial scale, presenting challenges for broader application. Consequently, there's ongoing research aimed at consolidating both catalysts within a single reactor to simplify the process for industrial applications.

#### Fixed bed reactor

The team of Castaño^160^ developed a fixed-bed reactor that was distinctively filled with two types of catalysts: one for the oligomerization of ethylene (Ni supported on H–Y zeolite) and another for acid-catalyzed reactions (ZSM-5). These catalysts were strategically placed in various configurations within the reactor, allowing them to operate under uniform pressure and temperature conditions. It was observed that a sequential arrangement of the catalysts—where they were not physically mixed but arranged in series—yielded superior results in terms of ethylene conversion and the selectivity for hydrocarbons in the jet fuel range compared to configurations where the catalysts were mixed. This innovative approach underscores the benefits of dual-catalyst systems in enhancing the efficiency and selectivity of chemical conversions for the production of jet fuels.

#### Hydrogenation of olefins

After the synthesis of olefins, they must undergo hydrogenation to be converted into paraffins. This step, carried out in the hydrogenation unit, is a widespread and optimized process in the petrochemical industry aimed at saturating the remaining double bonds in the olefins following oligomerization. The production of a sufficiently saturated product is essential to reduce the fuel's reactivity. Upstream of it, the hydrogenation process employs a solid catalyst.

## Outlook and conclusions

The Modified Fischer–Tropsch Synthesis (FTS) for the production of SAFs presents significant potential due to its ability to produce a wide range of hydrocarbons directly from CO_2_. However, the key challenge lies in achieving selectivity toward jet fuel-range hydrocarbons (C_8_–C_16_). The development of catalysts that can efficiently promote carbon chain growth while minimizing the production of undesired byproducts such as methane and light olefins is crucial. Additionally, the complexity of catalyst design, particularly in balancing the RWGS and FTS reactions, adds another layer of difficulty. Advances in catalyst formulation, such as the incorporation of promoters like potassium or the use of bimetallic systems, have shown promise, but achieving industrial scalability remains a challenge. Future research should focus on optimizing catalyst stability and selectivity, and on integrating these systems into larger-scale processes.

The synthesis of methanol from CO_2_ as an intermediate for SAF production is a critical pathway in the transition towards a sustainable aviation fuel industry. The current state of methanol synthesis technology. The development of catalysts with high activity and selectivity under CO_2_ hydrogenation conditions, particularly in the presence of impurities typically found in industrial CO_2_ streams, remains a significant challenge. As the energy transition progresses, innovations in catalyst design, such as the development of indium–cobalt systems, offer the potential to overcome these challenges by providing higher conversion efficiencies and stability under industrial conditions.

The methanol to olefins (MTO) process is a promising route for converting methanol into olefins, which can then be oligomerized to form hydrocarbons suitable for SAFs. Advances in catalyst design, particularly the development of shape-selective catalysts like ZSM-5 and SAPO-34, have significantly improved the process. However, catalyst deactivation due to coke formation remains a persistent issue. Future research should aim to enhance the lifetime of MTO catalysts through the development of hierarchical structures or the incorporation of mesoporosity, which could improve diffusion and reduce coke buildup.

The conversion of methanol to aromatics (MTA) is essential for producing the aromatic components of SAFs. The primary challenge in the MTA process is the development of catalysts that can achieve high selectivity towards the desired aromatics (benzene, toluene, and xylene) while minimizing the formation of undesired byproducts. Metal-modified ZSM-5 catalysts, particularly those incorporating Zn and Ga, have shown promise in enhancing aromatic yields. However, issues such as catalyst deactivation due to coke formation and the need for precise control of reaction conditions to optimize aromatic selectivity remain significant hurdles. Future research should focus on the development of more robust catalysts, possibly through the introduction of secondary mesoporosity or the use of novel metal-modification techniques.

To chart the future of aviation fuel derived from olefin oligomerisation, a well-conceived, efficient and sustainable methodology must be devised. The road ahead is fraught with challenges to overcome for practical implementation.

On the catalyst front, versatility is a major hurdle. While much research has been done on the oligomerisation of typical model molecules such as ethylene or propylene, the literature is sparse when it comes to the co-oligomerisation of various alkenes. This capability is crucial for producing jet fuel from methanol, as the MTO process produces a variety of olefins, which ideally should be co-oligomerised without prior separation. Moreover, co-oligomerisation of different olefins could lead to a mixture of higher olefins with significant molecular branching, a potential that Sauer *et al.*^185^ have already illustrated.

Catalyst longevity: improving the stability of catalysts is essential for their potential application. Oligomerisation of olefins on acid catalysts such as zeolites (essential for this process) is often accompanied by zeolite deactivation. This deactivation is usually due to the formation of aromatics and eventually coke. The literature shows that the use of mesoporous zeolites extends the lifetime of these catalysts and prevents heavier compounds from remaining within the zeolite porosity and acting as a coke precursor. Thus, methods such as desilication or the preparation of zeolite nanocrystals for the preparation of macro–mesoporous materials need to be further developed and studied.

Last but not least, process^155^ design and intensification are a must. On one hand, minimizing separation steps is crucial for the final carbon footprint of SAFs, hence smart process design together with more selective catalysts can really make a difference. On the other hand, process intensification through heat integration and combination of several steps within single reactor units can contribute to much more energy efficient processes.

## Data availability

No primary research results, software or code have been included and no new data were generated or analysed as part of this review.

## Author contributions

Conceptualization: Jorge Gascon, Jean Marcel R. Gallo, Jose L. Santos, and Enrique V. Ramos-Fernandez. Validation: Jorge Gascon. Resources: Enrique V. Ramos-Fernandez, Jose L. Santos, Dina K. Alsaadi, Anastasiya Bavykina and Jean Marcel R. Gallo. Writing (Original Draft): Enrique V. Ramos-Fernandez, Jose L. Santos, Dina K. Alsaadi, Anastasiya Bavykina and Jean Marcel R. Gallo. Writing (Review & Editing): Enrique V. Ramos-Fernandez, Jose L. Santos, Jean Marcel R. Gallo and Jorge Gascon. Visualization: Enrique V. Ramos-Fernandez, Jose L. Santos and Jorge Gascon. Supervision: Jorge Gascon.

## Conflicts of interest

There are no conflicts to declare.
